# Gout in the UK and Germany: prevalence, comorbidities and management in general practice 2000–2005

**DOI:** 10.1136/ard.2007.076232

**Published:** 2007-11-02

**Authors:** L Annemans, E Spaepen, M Gaskin, M Bonnemaire, V Malier, T Gilbert, G Nuki

**Affiliations:** 1 IMS Health, Brussels, Belgium; 2 Department of Public Health, Ghent University, Ghent, Belgium; 3 School of Pharmacy, Brussels University, Brussels, Belgium; 4 IMS Health, London, UK; 5 Ipsen, Paris, France; 6 Ipsen, Milford, Massachusetts, USA; 7 University of Edinburgh Rheumatic Diseases Unit, Western General Hospital, Edinburgh, UK

## Abstract

**Objective:**

To investigate and compare the prevalence, comorbidities and management of gout in practice in the UK and Germany.

**Methods:**

A retrospective analysis of patients with gout, identified through the records of 2.5 million patients in UK general practices and 2.4 million patients attending GPs or internists in Germany, using the IMS Disease Analyzer.

**Results:**

The prevalence of gout was 1.4% in the UK and Germany. Obesity was the most common comorbidity in the UK (27.7%), but in Germany the most common comorbidity was diabetes (25.9%). The prevalence of comorbidities tended to increase with serum uric acid (sUA) levels. There was a positive correlation between sUA level and the frequency of gout flares. Compared with those in whom sUA was <360 μmol/l (<6 mg/dl), odds ratios for a gout flare were 1.33 and 1.37 at sUA 360–420 μmol/l (6–7 mg/dl), and 2.15 and 2.48 at sUA >530 μmol/l ( >9 mg/dl) in the UK and Germany, respectively (p<0.01).

**Conclusions:**

The prevalence of gout in practice in the UK and Germany in the years 2000–5 was 1.4%, consistent with previous UK data for 1990–9. Chronic comorbidities were common among patients with gout and included conditions associated with an increased risk for cardiovascular disease, such as obesity, diabetes and hypertension. The importance of regular monitoring of sUA in order to tailor gout treatment was highlighted by data from this study showing that patients with sUA levels ⩾360 μmol/l (⩾6 mg/dl) had an increased risk of gout flares.

Gout is a disorder of purine metabolism characterised by acute, recurrent attacks of crystal arthritis. The single most important risk factor for developing gout is a raised level of serum uric acid (sUA), with supersaturation of uric acid in the extracellular fluid resulting in the precipitation of urate crystals.^1^ The 5-year cumulative risk of developing gout is 30.5% in men with an sUA level ⩾590 μmol/l (⩾10 mg/dl) and only 0.6% in those with an sUA level <420 μmol/l (<7.0 mg/dl).^2^ Deposition of urate crystals in the articular, periarticular and subcutaneous tissues^3^ results in episodes of acute arthritis (usually initially affecting the metatarsophalangeal joints) and the development of tophi.^4^ In addition, deposition of urate crystals in the renal tract may lead to impaired renal function.

The definitive diagnosis of gout depends on identifying monosodium urate crystals in fluid from aspiration of an acutely affected joint. However, joint aspiration and crystal identification by polarising light microscopy is generally regarded as a specialist procedure which is seldom undertaken in general practice, where most patients with gout are diagnosed and treated.^5^ ^6^ The diagnosis is usually made clinically, and often in retrospect, based on clinical symptoms described by patients, and on response to treatment.

The principal goal of treatment in chronic gout is to prevent crystal formation and promote crystal dissolution. Recent recommendations from the European League Against Rheumatism (EULAR)^7^ state that this is most effectively achieved by reducing and maintaining sUA levels below 360 μmol/l (6 mg/dl). Several studies provide evidence that reduced sUA is associated with reduced frequency of gout flares and reduction in tophi size.^3^ ^4^ ^8^ ^9^ Effective management requires long-term administration of uric acid lowering treatment, initially guided by, and then monitored through, regular sUA testing.

A recent study in the UK demonstrated the overall prevalence of gout to be 1.4%. Gout prevalence increased with age and was much higher among men.^10^ That study apart, however, contemporary data on the prevalence of gout in Europe are lacking, possibly because the episodic and chronic nature of the condition makes these data difficult to collect.

We therefore undertook a study to investigate the prevalence of gout, its comorbidities, and its current clinical management in general practice in the UK and Germany.

## METHODS

A retrospective analysis was conducted using the IMS Disease Analyzer, a longitudinal database containing anonymised patient records maintained by 650 general practitioners (GPs) treating 2.5 million patients in the UK and 400 GPs and internists treating 2.4 million patients in Germany.

Patients were included in the analysis if they had had a consultation with a diagnosis of gout (International Classification of Diseases (ICD-10) code M10 or “gout” written in the patient notes) between January 2000 and June 2005, and there was at least one additional recording of gout in their history (consultation with a diagnosis of gout or prescription for gout treatment). Patients were also required to have at least 24 months of recorded data before, and 18 months after, their index date (defined as their first consultation for gout between January 2000 and June 2005). Patients were excluded from the analysis if they were under 18 years of age or had been diagnosed with cancer.

The data analysed included comorbidities, medication use, sUA level and frequency of flares in patients with gout.

The following definitions were used for the purposes of identifying comorbidities: renal insufficiency (ICD-10 codes K767, N170, N171, N172, N178, N179, N180, N188, N189, N190 or I120; or a serum creatinine level of >150 μmol/l (>2 mg/dl)); alcoholism (ICD-10 codes F10, Y91, Y90 or K70); diabetes (a prescription for any antidiabetic drug; or ICD-10 codes E10, E11, E12, E13 or E14); heart failure (ICD-10 code I500, I501 or I509); hypertension (ICD-10 codes I100, I150, I152, I158, I159 or I270); myocardial infarction (ICD-10 codes I210, I211, I212, I213, I214, I219, I220, I221, I228, I229 or I252); and obesity (body mass index (BMI) >30 kg/m^2^; or ICD-10 codes E660, E661, E662, E668 or E669).

Persistence was defined as the total time that any patient was prescribed a given drug, from initiation of treatment to the end of the last supplied prescription, without intervening discontinuation of that drug.

Compliance was defined as the percentage of the prescribed doses that could actually have been taken by a patient during the period of persistence. Compliance was assessed by dividing the sum of the number of days of medication supplied by the duration of treatment while the patient was persistent (ie, the medication possession ratio).^11^

A gout flare was defined as either a visit resulting in a prescription for treating acute gout (short-term prescription for non-steroidal anti-inflammatory drugs (NSAIDs), colchicine or corticosteroids) or a gout-related emergency-room visit or admission to hospital.

Mean and standard deviation were determined for all variables during the analysis. A logistic regression model was applied to investigate the relationship between sUA and frequency of gout flares. The outcome assessed was the total number of recorded flares during the observation period (±3–4 years), in relation to the maximum observed sUA level in that period. Persistence was analysed using Cox regression analysis, which models the risk of treatment discontinuation over time.

## RESULTS

### Gout prevalence

During the observation period, the IMS Disease Analyzer contained 2 514 806 and 2 402 185 patient records in the UK and Germany, respectively. Overall, there were 34 071 patients in the UK and 34 797 in Germany who were reported to have gout, indicating a prevalence of 1.4% in both countries. On the basis of the inclusion and exclusion criteria, 7443 patients in the UK and 4006 in Germany were entered into the analysis (table 1). Over 80% of the study population was male in each of the countries. The mean age of the patients sampled was 66 years and 63 years in the UK and Germany, respectively, and the mean duration of gout history was 81 and 67 months, respectively.

**Table 1:** Study population characteristics

Characteristics	UK(n = 7443)	Germany(n = 4006)
Male patients, No (%)	6074 (81.6)	3222 (80.4)
Current age (years)	65.6 (13.8)	63.1 (13.1)
Age at diagnosis (years)	61.6 (13.9)	58.6 (13.1)
Duration of gout (months)	81.4 (66.7)	67.4 (28.6)
Time of follow-up (months)	46.2 (14.4)	51.0 (14.5)

Results are shown as mean (SD) unless stated otherwise.

### Pre-index comorbidities

**Figure 1:**
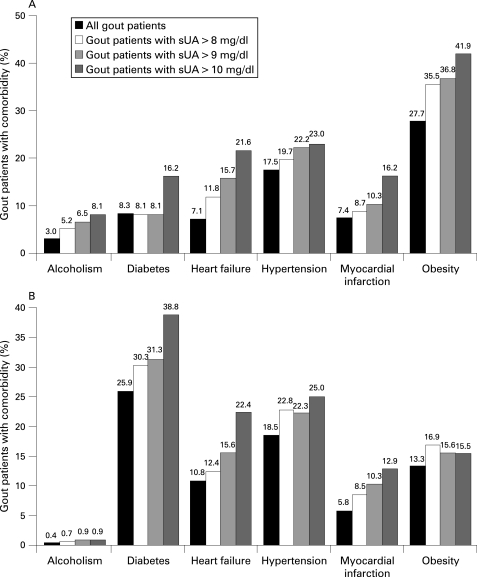
Pre-index comorbidities in (A) the UK study population and (B) the German study population. sUA, serum uric acid. Conversion: sUA (mg/dl) ×59.48  =  μmol/l.

Renal insufficiency was reported in 9.5% of the study population in the UK and 4.8% in Germany. Figure 1 shows the prevalence of other comorbidities within the study population. In the UK study population, the most common comorbidity recorded was obesity (27.7%), while in Germany it was diabetes (25.9%). Hypertension was a common comorbidity in both study populations: 18.5% of the German population sampled and 17.5% of the UK patients had at least one record of hypertension during the study period. Heart failure and myocardial infarction were reported in 7.1% and 7.4% of the UK study population, and 10.8% and 5.8% of the study population in Germany, respectively. Alcoholism was recorded as a diagnosis in 3% of UK patients with gout and 0.4% of the German patients with gout. There was a trend for the prevalence of each comorbidity to increase with sUA level (fig 1).

### Drug use before and after the index consultation

**Figure 2:**
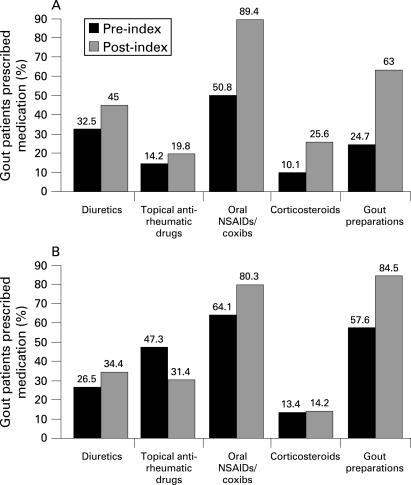
Drug use before and after the index consultation in (A) the UK study population and (B) the German study population. NSAIDs, non-steroidal anti-inflammatory drugs.

Figure 2 shows drug use before and after the index consultation in the UK and German study populations.

In the UK, 63% and in Germany 84.5% of patients with gout received treatment (fig 2). Among these patients, allopurinol was prescribed for most patients in both countries (89% UK; 93% Germany). Colchicine was used much less frequently (16% UK; 15% Germany). In the UK, the use of probenecid and sulfinpyrazone was limited to <1% of patients receiving gout pharmacological treatment. Benzbromarone was prescribed to <3% of patients in Germany. In addition to chronic gout treatment, 89.4% (UK) and 80.3% (Germany) of patients received prescriptions for oral NSAIDs as prophylaxis.

**Figure 3:**
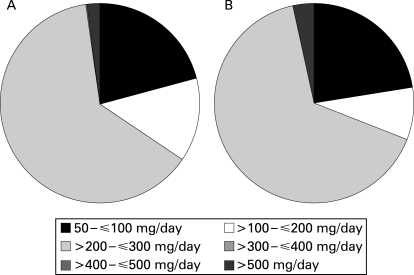
Average dosages of allopurinol prescribed in (A) the UK and (B) Germany.

Allopurinol was prescribed at an average daily dose of >200 mg or ⩽300 mg in 63.3% of patients in the UK and 65.7% of those in Germany (fig 3). Average daily doses in the range of 50–100 mg were prescribed in 21% of patients in the UK and 22.4% of those in Germany, and doses >300 mg/day were not often used (2.1% of patients in the UK and 3.4% of patients in Germany).

**Figure 4:**
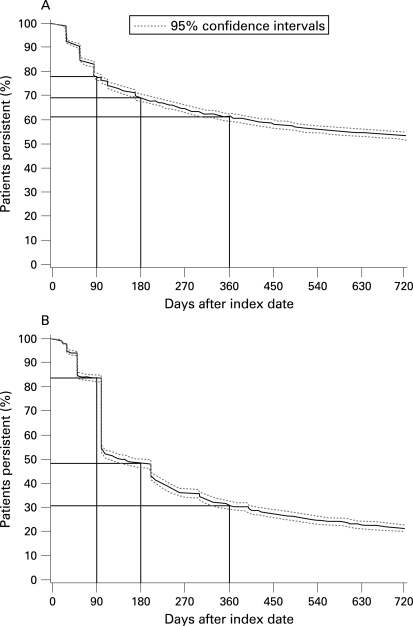
Persistence with allopurinol treatment at 24 months in (A) the UK study population and (B) the German study population.

In the UK, persistence with allopurinol (expressed as the percentage of patients receiving treatment for fixed time points) was 92.3% at 28 days, 76.3% at 100 days and 61.2% by 360 days (fig 4A). In Germany, persistence with allopurinol treatment was 98.0% at 28 days, 54.3% at 100 days and 31.0% by 360 days (fig 4B). Apparent rates of compliance while receiving treatment were 93% in the UK and 96% in Germany.

### sUA testing and gout flares

sUA testing was conducted during the observation period in 14% of patients in the UK and 9% of those in Germany. The proportion of patients who had one or more sUA tests performed annually during the observation period was <1% in both countries.

Overall, 72% of the study population in the UK and 41% in Germany experienced at least one gout flare during the observation period (table 2; 45.8%+26.2% and 17.2%+24.1%, respectively).The mean number of gout flares per patient was 1.7 in the UK and 2.9 in Germany (table 2). The frequency of gout flares was positively correlated with the level of sUA (table 2).

**Table 2:** Frequency of gout flares during the observation period. The observation period was unique for each individual patient and depended on when the index date occurred within the period January 2000–June 2005

	All patients		sUA* >7 mg/dl		sUA >8 mg/dl		sUA >9 mg/dl		sUA >10 mg/dl	
	UK	Germany	UK	Germany	UK	Germany	UK	Germany	UK	Germany
Patients with one gout flare during their observation period (%)	45.8	17.2	–	–	50.3	21.4	53.0	22.3	41.9	22.4
Patients with ⩾2 gout flares during their observation period (%)	26.2	24.1	–	–	42.8	33.3	47.0	31.3	58.1	30.2
Mean number of gout flares/patient	1.7	2.9	–	–	1.8	3.1	1.9	3.3	1.9	3.8
Mean number of gout flares/patient/year of observation period	0.38	0.68	0.42	0.65	0.43	0.67	0.42	0.91	0.58	1.97

Conversion: sUA (mg/dl) ×59.48  =  μmol/l.

sUA, serum uric acid.

In the UK, patients with an sUA of 360–420 μmol/l (6–7 mg/dl) were 1.33 times more likely to experience a gout flare than those in whom sUA was <360 μmol/l (<6 mg/dl; odds ratio (OR) = 1.33; p = NS), while those with an sUA level ⩾530 μmol/l (⩾9 mg/dl) were more than twice as likely (OR = 2.15; p<0.01) (table 3). In Germany, patients with an sUA level of 360–420 μmol/l (6–7 mg/dl) were 1.37 times more likely to experience a gout flare than those in whom sUA was <360 μmol/l (<6 mg/dl; OR = 1.37; p = NS), and those with an sUA level ⩾530 μmol/l (⩾9 mg/dl) were more than twice as likely to experience a gout flare (OR = 2.48; p<0.01) (table 3).

**Table 3:** Association between serum uric acid (sUA) level and the number of flares

sUA level* (mg/dl)	UK		Germany	
	Odds ratio vs sUA<6 mg/dl (CI)	p Value	Odds ratio vs sUA<6 mg/dl (CI)	p Value
6–7	1.33 (0.92 to 1.94)	NS	1.37 (0.91 to 2.05)	NS
>7–8	1.49 (1.21 to 2.42)	<0.01	1.65 (1.17 to 2.33)	<0.01
>8–9	1.71 (1.04 to 2.13)	<0.01	2.37 (1.67 to 3.36)	<0.01
⩾9	2.149 (1.53 to 3.01)	<0.01	2.48 (1.77 to 3.49)	<0.01

*Conversion: sUA (mg/dl) ×59.48  =  μmol/l.

## DISCUSSION

This study was undertaken to augment the limited data available on the epidemiology of gout and its management in practice in Europe. The study dealt exclusively with patients with a clinical diagnosis of gout but not patients with asymptomatic hyperuricaemia. The overall prevalence of gout of 1.4% was remarkably similar in two large IMS databases, each containing about 2.5 million patient records from the years 2000–5 in Germany and the UK. The overall prevalence was also remarkably similar to that found previously by Mikuls *et al*^10^ in the records of 1.8 million patients in the UK General Practice Research Database (GPRD) for the years 1990–9, using similar case definitions.^10^

The consistency of these observations on the prevalence of gout consultations in Germany and the UK would seem to provide a measure of confidence in the robustness of estimates of the true prevalence of gout in primary care settings in Northern Europe. It is noteworthy, however, that the prevalence of gout was not greater in the current study, than in the UK GPRD for 1990–9,^10^ despite the fact that the mean ages of the patients with gout in the UK and German databases for 2000–5 were 6 years and 3 years older, respectively. The prevalence rates observed are, however, slightly higher than those found by Harris *et al* (0.95%) in 40 UK general practices in 1993^12^ and fivefold higher than those observed (0.26%) in the survey by Currie in UK general practices 30 years ago.^13^ There are other data which suggest that the prevalence of gout might have increased over the past 30 years. The annual prevalence of self-reported gout in the National Health Interview Survey in the USA trebled between 1969 and 1996,^14^ and the prevalence of gout and hyperuricaemia requiring urate-lowering drug treatment was observed to have increased by 80% between 1990 and 1999 in a managed-care population in the USA.^15^ However, Mikuls *et al* showed that the overall incidence of consultations for gout remained relatively stable throughout the 1990s,^10^ suggesting that any increase in prevalence might be largely attributable to the changing age structure of the population. Nevertheless, gout is the most common cause of inflammatory arthritis in men over the age of 40 years,^16^ and the overall prevalence of gout is notably higher than that of rheumatoid arthritis, which was recently estimated to be 1.16% in women and 0.44% in men across the UK population.^17^

As expected, the prevalence of comorbidities was high among patients with gout in both the UK and Germany. The comorbidities found to be most commonly associated with gout in this study were obesity in the UK and diabetes in Germany. Obesity was twice as common (27.7%) in UK patients as in German patients (13.3%), while diabetes was recorded as a comorbid diagnosis three times as often in the German patients (25.9%) as in those in the UK GPRD (8.3%). Similar prevalence rates of obesity have been estimated previously in the UK and Germany,^18^ and the somewhat surprising difference reported in this study may be attributable, in part, to the use of the IMS Disease Analyzer database. In the UK, GPs in the IMS Disease Analyzer panel can record items such as BMI in addition to using ICD-10 codes for obesity, but in Germany only the ICD-10 codes were considered in the study definition of obesity as there was no other electronically coded way for a GP to record obesity. Therefore, it is possible that British GPs were more likely to capture information electronically about obesity than those in Germany. The greater frequency with which diabetes was recorded in Germany is consistent with previous estimates of prevalence rates in Europe, which were more than twice as high in Germany as those in the UK.^19^

Over 60 years ago, Brochner-Mortensen observed that 78% of a group of 100 Scandinavian patients with gout were more than 10% overweight and 57% were more than 30% overweight.^20^ Grahame and Scott found that 48% of a UK cohort of 354 patients with gout attending a hospital clinic were more than 15% overweight.^21^ Obesity was not recorded as a specific comorbidity in the study by Mikuls *et al*.^10^ However, recently published data demonstrated that the prevalence of abdominal obesity and metabolic syndrome is as high as 62.9% in people with gout identified in the US Third National Health and Nutrition Survey (NHANES III),^22^ and diabetes was a comorbid diagnosis in 19.9% of 9482 patients with gout in a North American study of patients in a managed-care setting.^23^

It is therefore interesting and surprising that Mikuls *et al* also found the prevalence of diabetes to be remarkably low (6.4%) in patients with gout in their analysis from the UK GPRD for 1990–9.^10^ In that study, it was found that coronary artery disease and hypertension were the most common comorbidities in patients with gout (24.9% and 23.9%, respectively).^10^ The prevalence of hypertension among patients with gout was found to be as high as 59.7% in the North American managed-care population.^23^

There is some evidence to suggest that gout and hyperuricaemia may be independent risk factors for cardiovascular disease.^24^ ^25^ The clinical data demonstrating the association of gout with obesity, hypertension, excessive alcohol consumption and metabolic syndrome is now overwhelming and incontrovertible.^22^ ^23^ ^25^ ^26^ As these comorbidities are themselves major risk factors for cardiovascular disease, every diagnosis of gout should act as a “red flag” to alert doctors to assess patients for cardiovascular risk.^7^ ^22^ ^27^

A similar proportion of patients were found to be receiving prescriptions for diuretic drugs in the UK and Germany in this study (∼ 30%), and by Mikuls *et al*.^10^ Diuretics, especially thiazide diuretics, can increase the sUA level as a consequence of volume depletion, increased renal tubular reabsorption of urate, stimulation of the urate-anion exchanger (URAT-1) and a decrease in renal urate excretion.^28^ Diuretic use has been found to be a risk factor for incident gout (relative risk (RR) = 1.77; 95% confidence interval 1.42 to 2.20) which is apparently independent of hypertension in the 12 years of follow-up of a cohort of men who had no previous history of gout in the health professionals follow-up study.^26^ However, a recent case–control study found no evidence that diuretics were a risk factor for the development of gout, and suggested that the cardiovascular conditions for which they were prescribed, which are themselves risk factors for gout, might have confounded previous inferences.^29^ This would imply that continuing flares of gout in these patients were not primarily attributable to continuing treatment with diuretics, but rather were a consequence of failure to treat hyperuricaemia adequately.

Allopurinol was by far the most commonly used drug for treating patients with chronic and interval gout in both the UK and Germany, accounting for about 90% of all such medication. However, despite the clear preference for allopurinol as the preferred uric acid lowering drug, the findings from the current study show that the management of patients with gout is suboptimal. A large proportion of patients with gout continued to have raised sUA levels and recurrent gout flares. Persistence of treatment with allopurinol was remarkably low in both the UK and Germany, but this study provides no information about why this should have been the case.

Until recently, the importance of follow-up sUA testing to guide treatment decisions in patients diagnosed with gout has not been emphasised or well defined by research evidence. However, there is now evidence from comparative randomised controlled trials,^3^ ^30^ and from an observational study in a managed-care setting,^1^ that fewer than 50% of patients receiving allopurinol, in the most commonly prescribed dose of 300 mg daily, achieve optimum reductions in plasma urate concentrations. Recent evidence-based, consensus guidelines from EULAR^7^ emphasise the importance of ensuring that the sUA level is maintained below 360 μmol/l (6 mg/dl), but those from the British Society for Rheumatology^27^ recommend maintaining the sUA level below 300 μmol/l (5 mg/dl). Despite a lack of evidence for the optimum frequency for monitoring the sUA level, recent guidelines recommend 3-monthly measurements of sUA in the first year after the start of treatment with uric acid lowering drug treatment followed by annual measurements.^27^ The frequency of sUA testing was found to be extremely low in this study; sUA evaluation was performed in only 14% of the UK and 9% of the German study populations, and fewer than 5% of patients had two sUA tests performed in the observation period in either country. It might be argued that it is acceptable for the sUA level to be monitored less frequently in patients with good symptomatic control; however, the study clearly showed that even patients who continued to experience gout flares were not having regular sUA checks.

Seventy-two percent of the patients with gout in the UK, and over 40% of those in Germany, experienced at least one gout flare, and the frequency of gout flares was significantly correlated with sUA levels.

Previous studies have demonstrated the presence of urate crystals in the joints of patients with asymptomatic gout,^31^ but reduction of the sUA below 360 μmol/l (6 mg/dl) leads to decreased intra-articular crystal deposition^8^ and more rapid reduction of tophi^3^ as well as a reduction in the frequency of gout flares.^9^ This suggests that maintaining the sUA level below 360 μmol/l (6 mg/dl) could prevent recurrent attacks of acute gout in the long term.^1^ ^9^ Indeed, it would be of interest to undertake further research to investigate the possibility of a continuous relationship between sUA levels and the risk of crystal deposition, analogous to the relationship that has been demonstrated between cardiovascular risk and measures such as serum cholesterol levels and blood pressure.

This study has some limitations. Although the primary care consultation data provided by the IMS databases are an important source of information on the prevalence of gout, associated comorbidities and treatment, and can provide age and sex standardised estimates of the number of new consultations, the IMS Disease Analyzer does not contain community-based data that would allow for the measurement of population-wide incidence rates of gout in different countries. Although the IMS database populations in Germany and the UK closely mirror the populations at large in their male to female ratio, social class and geographical distribution, it is possible that not all practice types are represented.

Possibly, the study might have underestimated the prevalence of gout in Germany, as several patients in the German database were excluded from the analysis because they only had a single recording of gout in their notes. Second attacks might have been under-recorded in the German database because of the free choice of healthcare that operates in Germany, where the doctor responsible for the patient’s records might not be the doctor consulted for a subsequent gout flare. There is also the possibility that the study underestimated the frequency of gout flares, because the option to self-medicate means not all gout flares would necessarily have been detailed in a patient’s records. Any such effect did not, however, prevent the study from demonstrating a correlation between sUA level and gout flares.

Another important question that was not dealt with was whether the doses of allopurinol were optimum in those patients with gout found to have a high sUA level and frequent gout flares. The study showed that 97.9% of patients prescribed allopurinol in the UK and 96.6% of those in Germany received doses of ⩽300 mg/day (fig 3), but how this was related to outcomes was not investigated. Neither did the study evaluate whether allopurinol doses were adjusted according to renal function to minimise the risk of toxicity in patients with renal impairment.^27^ ^32^

The relationship between flares and the recommended^7^ ^27^ prophylactic use of low-dose colchicine or NSAIDs to minimise the risk of gout flares following the initiation of urate-lowering treatment was not investigated.

In conclusion, this study has shown that the prevalence of gout in practice in the UK and Germany in the years 2000–5 was 1.4%, a figure that is consistent with previous UK data for 1990–9. Patients with gout had a high frequency of chronic comorbidities, several of which are associated with an increased risk of cardiovascular disease. Allopurinol was the preferred urate-lowering drug for the management of interval and chronic gout in both countries studied, but persistence with treatment was poor and gout flares were frequent despite urate-lowering treatment.

Evaluation of sUA levels to guide treatment was not undertaken sufficiently. The importance of regular monitoring of sUA levels and adjustment of treatment to optimise sUA was highlighted by data from this study, showing that the prevalence of comorbidities among patients with gout was directly related to sUA levels, and that patients with sUA levels >360 μmol/l (>6 mg/dl) had an increased risk of gout flares.
